# Myocardial infarction stabilization by cell‐based expression of controlled Vascular Endothelial Growth Factor levels

**DOI:** 10.1111/jcmm.13511

**Published:** 2018-02-25

**Authors:** Ludovic Melly, Giulia Cerino, Aurélien Frobert, Stéphane Cook, Marie‐Noëlle Giraud, Thierry Carrel, Hendrik T. Tevaearai Stahel, Friedrich Eckstein, Benoît Rondelet, Anna Marsano, Andrea Banfi

**Affiliations:** ^1^ Cell and Gene Therapy Departments of Biomedicine and Surgery University and University Hospital Basel Basel Switzerland; ^2^ Cardiac Surgery and Engineering Departments of Biomedicine and Surgery University and University Hospital Basel Basel Switzerland; ^3^ Department of Cardiac Vascular and Thoracic Surgery CHU UCL Namur Yvoir Belgium; ^4^ Department of Cardiology University of Fribourg Fribourg Switzerland; ^5^ Department of Cardiovascular Surgery, Inselspital Bern University Hospital and University of Bern Bern Switzerland

**Keywords:** myocardial infarction, adipose stem cells, Vascular Endothelial Growth Factor, angiogenesis, cell therapy

## Abstract

Vascular Endothelial Growth Factor (VEGF) can induce normal or aberrant angiogenesis depending on the amount secreted in the microenvironment around each cell. Towards a possible clinical translation, we developed a Fluorescence Activated Cell Sorting (FACS)‐based technique to rapidly purify transduced progenitors that homogeneously express a desired specific VEGF level from heterogeneous primary populations. Here, we sought to induce safe and functional angiogenesis in ischaemic myocardium by cell‐based expression of controlled VEGF levels. Human adipose stromal cells (ASC) were transduced with retroviral vectors and FACS purified to generate two populations producing similar total VEGF doses, but with different distributions: one with cells homogeneously producing a specific VEGF level (SPEC), and one with cells heterogeneously producing widespread VEGF levels (ALL), but with an average similar to that of the SPEC population. A total of 70 nude rats underwent myocardial infarction by coronary artery ligation and 2 weeks later VEGF‐expressing or control cells, or saline were injected at the infarction border. Four weeks later, ventricular ejection fraction was significantly worsened with all treatments except for SPEC cells. Further, only SPEC cells significantly increased the density of homogeneously normal and mature microvascular networks. This was accompanied by a positive remodelling effect, with significantly reduced fibrosis in the infarcted area. We conclude that controlled homogeneous VEGF delivery by FACS‐purified transduced ASC is a promising strategy to achieve safe and functional angiogenesis in myocardial ischaemia.

## Introduction

Ischaemic heart pathologies are caused by inadequate balance between myocardial oxygen supply and demand such as in angina or myocardial infarction. Percutaneous coronary intervention (PCI) and coronary artery bypass grafting (CABG) typically restore the macrovascularization in the ischaemic area of the either acutely or chronically affected myocardium. But many patients could also benefit from additional therapeutic angiogenic strategies that would improve the microvascularization 1.

VEGF is the master regulator of angiogenesis 2, but it can also induce aberrant and dysfunctional vascular growth if its expression is not tightly controlled 3, 4. We previously found that, as VEGF binds tightly to extracellular matrix, the induction of normal or aberrant angiogenesis depends strictly on its amount localized in the microenvironment around each producing cell and not simply on the total dose delivered 5, 6. This finding helps explaining the apparently narrow therapeutic window for VEGF gene therapy and its lack of efficacy at safe vector doses in first‐generation clinical trials for therapeutic angiogenesis 7. In these trials, only the total dose of vector could be controlled, but not the level of expression achieved in individual cells, leading to a heterogeneous and unpredictable distribution of VEGF within the tissue 8.

To overcome this limitation and the ensuing loss of therapeutic potential, we previously developed a high‐throughput, FACS‐based technology to identify and rapidly purify cells expressing a specific desired VEGF level from a heterogeneous population of transduced progenitors. This was achieved using a retroviral construct expressing the VEGF sequence quantitatively linked through an internal ribosome entry site (IRES) to a FACS‐quantifiable syngenic cell surface marker (truncated CD8a) so that the amount of CD8 on the cell surface would reflect the level of secreted VEGF 9. Populations of so‐purified VEGF‐expressing primary myoblasts could ensure robust, normal and stable angiogenesis both in normal 9 and chronically ischaemic 10 skeletal muscle, while angioma growth was completely avoided. However, skeletal myoblasts are not suitable for cardiac cell therapy, because of their inability to electrically couple and integrate with the surrounding cardiomyocytes, leading to fatal arrhythmias 11. On the other hand, adipose tissue‐derived stromal cells (ASC) possess several desirable features for cell therapy approaches to cardiac repair 12. In fact, ASC (*i*) can be easily obtained in large quantities from autologous liposuction material 13, (*ii*) have differentiation potential into cardiomyocytes 14 and (*iii*) have been reported to reduce the infarct area 15 and to significantly improve the left ventricular ejection fraction 16, as well as the electrophysiological function after delivery in animal models of myocardial infarction 12. Recently, we have shown that injection of genetically engineered VEGF‐expressing human ASC in normal myocardium, FACS purified to homogeneously produce a specific controlled and safe level of VEGF, could induce normal and stable angiogenesis while completely preventing aberrant vascular growth 17.

In this study, we tested the hypothesis that delivery of human ASC FACS purified to express controlled VEGF levels at the infarct border zone could efficiently induce safe angiogenesis and ultimately improve cardiac function in a rat model of myocardial infarction.

## Materials & methods

### Cell culture

Human adipose tissue was obtained from three healthy patients undergoing plastic surgery after informed consent and according to a protocol approved by the Ethical Committee of the Basel University Hospital. All investigations conformed to the Declaration of Helsinki. Tissue was minced and digested with 0.15% collagenase (Worthington Biochemical Corporation, Lakewood, NJ) in phosphate‐buffered saline (PBS) at 37°C under continuous shaking for 60 min. After centrifugation at 1500 rpm for 10 min, the lipid‐rich layer was discarded and the cellular pellet was washed once with PBS. Released cells were strained through a 100‐μm nylon‐mesh followed by a 70‐μm nylon‐mesh to remove fibrous debris, plated at a density of 10^5^ cells/cm^2^ and cultured in high‐glucose Dulbecco's modified Eagle's medium with 10% foetal bovine serum, as described 18. After 4 days, cells were retrovirally transduced twice a day according to a recently optimized high‐efficiency protocol 19 for a total of six rounds. The retroviral vectors expressing rat VEGF_164_ and truncated rat CD8a have been previously described 10. We have previously found that neither the transduction procedure nor VEGF expression affected the surface marker expression profile, proliferative ability and multi‐lineage differentiation potential of the transduced ASC 19. After 12 days, (approximately 90% confluence), cells were FACS‐sorted with a BD Influx Cell Sorter (BD Biosciences, Basel, Switzerland) to generate the three different treatment groups (*CD8, ALL and SPEC*), according to a previously published protocol 9, 17. Briefly at the first passage, control cells, which were transduced with the control virus expressing CD8 alone, were FACS purified to eliminate the few non‐transduced cells and yield a purely CD8‐positive population (CD8). Part of the cells transduced with the virus containing VEGF were FACS purified through a specific narrow gate to produce a homogeneous cell population expressing a specific moderate VEGF level (SPEC) and the rest of the cells were sorted to generate a population expressing all the heterogeneous VEGF levels (ALL).

### VEGF ELISA measurements

The production of rat VEGF_164_ and human VEGF_165_ was quantified as previously described 17. Briefly, species‐specific Quantikine VEGF ELISA kits (R&D Systems, Abingdon, UK) were used according to manufacturer's protocol. One millilitre of medium was harvested from ASC cultured in a 60‐mm dish, following 4 hrs of incubation, filtered and analysed in duplicate. Results were normalized by the number of cells and time of incubation and expressed as ng of VEGF/10^6^ cells/day. Four separate dishes of cells were assayed for each cell type from three independent donors.

### Animals

All animals were treated in compliance with Swiss Federal guidelines for animal welfare and all procedures were approved by the Veterinary Office of the Canton Bern (Bern, Switzerland) and conform to the Directive 2010/63/EU of the European Parliament. Immunodeficient male nude rats (Hsd: RH‐rnu/rnu, Harlan, Venray, Netherlands) were used to prevent rejection of human ASC.

### Myocardial infarction and *in vivo* cell implantation

Anaesthesia was performed with isoflurane (5% of oxygen for induction and 2.5% for maintenance) and additional buprenorphine (10 mg/kg). Animals were placed on a warming pad (37°C) and intubated with a 14G tracheal cannula (Abbocath, Abbott, Sligo, Ireland) and ventilated at 80 cycles/min (Small Animal Ventilator 683, Harvard Apparatus, Inc., Holliston, MA, USA). Hearts were exposed through a left thoracotomy 20. After opening the pericardium, a myocardial infarction was created by a permanent ligation of the left anterior descending (LAD) coronary artery using a 7/0 polypropylene suture. Distal ligature allowed the induction of an initial small infarct with limited mechanical overload and consequently reduced animal mortality over the study period. Two weeks after coronary ligation, a pre‐treatment echocardiography (E1) was performed to exclude animals with an ejection fraction above 60% (*i.e*. with no significant infarction) or under 45% (*i.e*. already close to heart failure), before randomization to one of the four treatment groups. Animals received five direct intramyocardial injections into the anterior wall around the infarction for a total of 150 μl of either PBS alone or containing 10^7^ cells for each of the three selected conditions (*CD8, ALL and SPEC*). Four weeks post‐treatment, rats were killed by exsanguination under the same anaesthesia conditions as already described, and the hearts were then collected, embedded in OCT compound (Sakura Finetek, Torrance, CA, USA) and frozen in isopentane cooled in liquid nitrogen.

### Functional assessment

Before killing at 28 days, animals were anaesthetized by mask and placed on the left lateral position. A single examiner evaluated left ventricular function in a blinded manner by transthoracic echocardiography equipped with a 9–11 MHz linear phase array transducer system (AcusonSequoia, Siemens, Inc., Malvern, PA). Echocardiographic assessments were performed on animals under anaesthesia induced by 2.5% isoflurane at the beginning of the study in non‐infarcted heart (E0), 2 weeks after induction of the myocardial infarction at the time of randomization (E1) and finally 4 weeks after treatment (E2). The fraction shortening (FS) and ejection fraction (EF) were recorded with M‐mode and 2D echocardiography. For each data point, three different heart cycles were averaged.

### Immunofluorescence

Histological analyses were performed on heart 10 μm‐thick cryosections. Immunofluorescence was performed using the following primary antibodies and dilutions: mouse anti‐rat CD31 (Clone TLD‐3A12; AbD Serotec, Düsseldorf, Germany; 1:100), rabbit antimouse NG2 (Millipore, Zug, Switzerland; 1:200), mouse anti α‐smooth muscle actin (Clone 1A4; MPBiomedicals, Basel, Switzerland; 1:400), anti‐human leucocyte antigen (HLA) (Biolegend, San Diego, CA, USA; 1:200), mouse anti‐Human Nuclei (HuNu) (Millipore, Billerica, MA, USA; 1:100), goat anti‐VE‐Cadherin (VE‐Cad; Santa Cruz, Dallas, TX, USA; 1:100) and goat anti‐rat VEGF (R&D Systems; 1:100). Fluorescently labelled Alexa488, Alexa546 or Alexa647 secondary antibodies (Invitrogen, Basel, Switzerland) were used at 1:200.

### Fibrosis evaluation

Histological analyses were performed on heart cryosections stained with Masson's Trichrome according to standard protocols. Images were acquired using an Olympus BX61 microscope (Olympus, Münster, Germany) and processed using Image J (National Institutes of Health, Bethesda, MD, USA) by colour recognition. In all experimental groups, the total amounts of fibrotic tissue (green colour) and muscle tissue (red tone) were determined on a standardized set of 10 cross‐sections, starting at the level of the papillary muscle and moving towards the apex every 300 μm. The percentage of fibrosis was calculated from the ratio of the green area over the total (red + green) area.

### Vessel measurements

The amount of blood vessels was quantified on images taken from CD31‐stained sections. Eighteen representative fluorescent images per group (six images/heart and three hearts/group) were acquired with a 20× objective on an Olympus BX61 microscope (Olympus) on cross‐sections of the anterior wall taken at 300‐μm intervals starting from the level of the papillary muscle, in the area where injections were performed. Blind analysis was performed with either Image J software (National Institutes of Health) or AnalySIS D software (Soft Imaging System, Münster, Germany). The area fraction occupied by endothelial cells was calculated by measuring the number of CD31‐positive pixels in each image with Image J software and normalizing it to the total area. Alternatively, vessel length density (VLD) was quantified as previously described 21 by tracing the total length of CD31‐positive vessels in AnalySIS D software (Soft Imaging System) and dividing it by the total area of each field.

### Statistics

Data are presented as means ± standard deviation and were analysed with the statistical software Prism 5.0a (GraphPad, La Jolla, CA, USA). For quantifications of VEGF expression, cardiac fibrosis and vascularization, comparison between groups was performed with the non‐parametric Kruskal–Wallis test for multiple comparisons and Dunn's post‐hoc test. All quantifications were analysed using the means of multiple individual measurements in each sample (*n* = number of independent samples). Echocardiography data, which represent measurements from the same animals before and after treatment, were analysed by Repeated Measures anova and Bonferroni post‐hoc test. The normal distribution of data sets representing differences between pre‐ and post‐treatment (∆EF and ∆FS) was verified and multiple comparisons were performed with the parametric 1‐way analysis of variance (anova) followed by the Bonferroni test. Differences were considered statistically significant when *P *<* *0.05.

## Results

**Figure 1:**
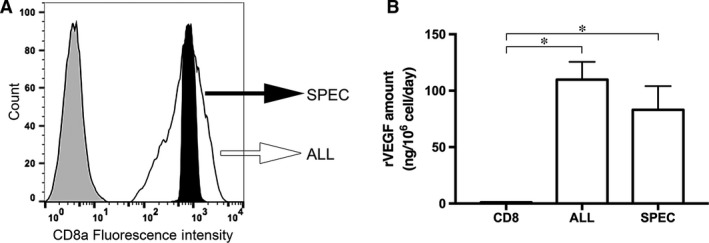
Cell generation and VEGF quantification. (**A**) VEGF‐expressing ASC were FACS‐sorted to generate two populations producing either a specific homogenous level (SPEC) or all heterogeneous levels (ALL) of VEGF. In the FACS plots: grey tinted curve = negative control; black open curve = purified ALL cells; black tinted curve = purified SPEC cells. (**B**) ELISA quantification of rat VEGF production in the culture supernatants of the different populations, expressed in ng/106 cells/day; * *P* < 0.05.

VEGF‐producing ASC were generated by transduction with a retrovirus carrying a bicistronic cassette that co‐expresses rat VEGF_164_ and a truncated version of rat CD8a as a FACS‐quantifiable cell surface marker. The primary transduced cells were used to generate two distinct populations. Part were FACS purified through a specific narrow gate, designed according to a previously published technique 9, to produce a homogeneous cell population expressing a specific moderate VEGF level (SPEC; Fig. 1 **A**). The rest were sorted to eliminate only non‐expressing cells and generate a population heterogeneously expressing all the VEGF levels present after retroviral transduction (ALL; Fig. 1 **A**). The mesenchymal identity of the different ASC populations used here was previously confirmed by their robust expression of classical mesenchymal markers (CD73, CD90, CD105) and essentially absent expression of endothelial (CD31, CD34, VEGFR2) or cardiac (Troponin I) markers 17. Further, the ASC phenotype was not affected by retroviral transduction, FACS‐sorting or VEGF expression 17, 19. In agreement with this, we have also previously shown that both control CD8 and ALL VEGF‐expressing cells maintained their *in vitro* differentiation potential towards the adipogenic or osteogenic lineages compared to the naïve ASC 19. *In vitro* VEGF release by cells from the different groups was quantified before *in vivo* injection. As shown in Fig. 1 **B**, negative control CD8 cells, which were transduced with a retrovirus carrying only the surface marker CD8, but no VEGF gene, produced negligible amounts of rat VEGF (CD8 = 1.0 ± 0.3 ng/10^6^ cells/day). On the other hand, both VEGF‐expressing populations (SPEC and ALL) produced similar total amounts of rat VEGF (ALL = 109.8 ± 15.8 ng/10^6^ cells/day; SPEC = 83.1 ± 21.1 ng/10^6^ cells/day), in agreement with the fact that the purified SPEC population represents the middle portion of the levels present in the unpurified ALL population, which further comprises both higher and lower ones, as visible on the FACS distribution of fluorescence intensities (Fig. 1 **A)**. On the other hand, as ASC were of human origin, expression of the endogenous human VEGF was also quantified. All three populations secreted very low amounts of human VEGF_165_, without any difference between conditions (CD8 = 13.8 ± 5.2; SPEC = 15.6 ± 6.0; and ALL = 15.3 ± 4.8 ng/10^6^ cells/day). Lastly, neither the genetic modification of the cells nor their sorting affected their morphology, which was uniformly fibroblast‐like, typical of early‐passage ASC (Fig. S1).

Surgical induction of myocardial infarction (MI) caused a moderate 24‐hr mortality of 15.7% (11/70 rats). To ensure a homogeneous functional state before randomization to the different treatment groups, 20 rats (33.8% of the surviving animals) were excluded because the echocardiographic control after 2 weeks indicated either a very mild or no infarction, evidenced by a left ventricular EF above 60%, or on the contrary a too severe infarction zone with a corresponding EF under 45%. Therefore, a total of 39 animals weighing 252 ± 16 grams with an EF of 45‐60% were randomized into the four treatment groups. This very selective randomization of animals with only an ejection fraction within a narrow range was necessary to ensure the standardization of the model and homogeneous functional groups for comparison 20. Mortality following the second thoracotomy and the intramyocardial injection was 10.3% (4/39). No further mortality was observed until follow‐up was completed at 6 weeks. Finally, 35 animals could be analysed in the four treatment groups: PBS *n* = 7; CD8 *n* = 9; ALL *n* = 8; SPEC *n* = 11 (Table 1).

**Table 1:** Overview of experimental groups

	*n*	%
Number of animals initially included	70	
Mortality post‐infarction*	11	15.7
Drop‐out†	20	33.8
Mortality post‐treatment	4	10.2
Completed follow‐up @ 6 weeks		
Total	35	50.0
Phosphate‐buffered saline	7	
CD8	9	
ALL	8	
SPEC	11	

*During the first 24 hrs after coronary ligation; ^†^Criteria for a drop‐out before randomization to treatment were a left ventricular ejection fraction (EF) >60% or <45%.

### Controlled VEGF expression prevents cardiac function deterioration after infarction

**Figure 2:**
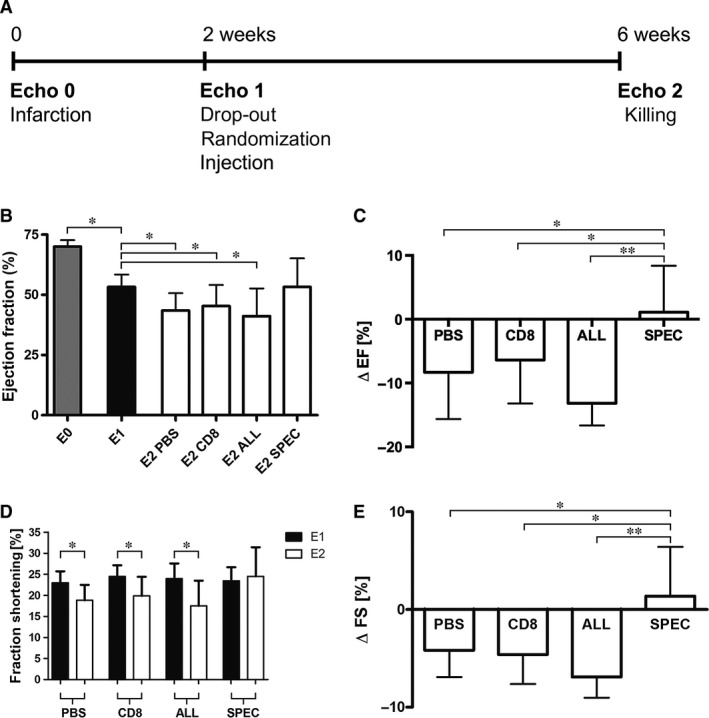
Echocardiographic cardiac functionality. (**A**) Time line of the experiment. Three echocardiography studies were performed at pre‐infarction (E0), 2 weeks after ligation of the left anterior descending but before treatment (E1) and 4 weeks post‐treatment (E2). Treatment consists in the injection of PBS, CD8 cells or VEGF ‐producing cells with controlled levels (SPEC) or heterogenous levels (ALL). Ejection fraction (2D‐mode) in non‐infarcted hearts and pre‐treatment, as well as post‐treatment per group (**B**) or expressed as difference between E1 and E2 per animal in the same group (**C**). Fraction shortening (M‐mode) per treatment group (**D**), or as a difference between E1 and E2 per animal of the same group (**E**). Phosphate‐buffered saline *n* = 7; CD8 *n* = 9; ALL *n* = 8; SPEC *n* = 11; * *P* < 0.05, ** *P* < 0.01.

Cardiac functionality was assessed before infarction (E0), 2 weeks after ligation of the LAD right before treatment (E1) and 4 weeks post‐treatment (E2) by echocardiography (Fig. 2 **A**). On day 14, E1 echocardiography analysis showed that at the time of randomization before treatment, the ejection fraction was significantly reduced compared to the non‐infarcted heart (E0) (53 ± 6% *versus* 70 ± 3%). After randomization, all groups had a similar EF before treatment with no statistical difference. Four weeks after treatment, EF further decreased in the PBS (−8 ± 7%), the CD8 (−6 ± 7%) and the ALL (−13 ± 10%) groups, but remained stable in the SPEC group (+1 ± 7%), (Fig. 2 **B**–**C**). Interestingly, EF data suggested a statistically non‐significant trend towards an even greater degree of deterioration after injection of cells expressing VEGF at uncontrolled levels (ALL) compared to PBS and control cells (CD8). Analysis of fractional shortening showed similar results (Fig. 2 **D**–**E)**, with a significant decrease between E1 and E2 for the PBS (−4 ± 3%), the CD8 (−4 ± 3%) and the ALL (−7 ± 6%) groups, but a stabilization for the SPEC treatment group (+1 ± 5%).

### Controlled VEGF expression reduces myocardial fibrosis

**Figure 3:**
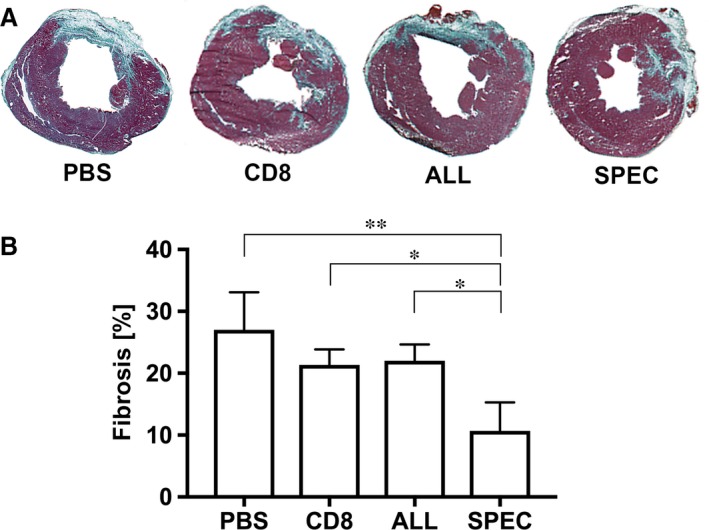
Fibrosis quantification. (**A**) Representative cryosections stained with Masson's Trichrome for the four treatment groups, corresponding to the median value of the quantification. (**B**) Fibrosis quantification measured in the percentage of green pixels over the total coloured pixel numbers for all treatment groups (CD8 and Phosphate‐buffered saline *n* = 3; ALL and SPEC *n* = 4); * *P* < 0.05, ** *P* < 0.01.

To assess the effects of the angiogenic treatment on ventricle remodelling, we evaluated the fraction of the left ventricular myocardium that was substituted by a scar tissue 6 weeks after coronary ligation. Masson's Trichrome staining of heart sections was used to detect the high collagen content typical of fibrotic scarring (Fig. 3 **A**, showing representative images corresponding to the median value of each group). In all the groups, fibrous tissue and a corresponding thinning of the regional wall thickness substituted a significant portion of the left ventricle. This was however greatly reduced only in the SPEC‐treated hearts. Quantification of the scar fraction (Fig. 3 **B**) confirmed that only controlled VEGF delivery (SPEC) could significantly reduce fibrosis (11 ± 5%) compared to all other groups (PBS = 27 ± 6%; ALL = 22 ± 3%; CD8 = 21 ± 3%), which were not statistically different from each other.

### Controlled VEGF expression improves normal myocardial angiogenesis

**Figure 4:**
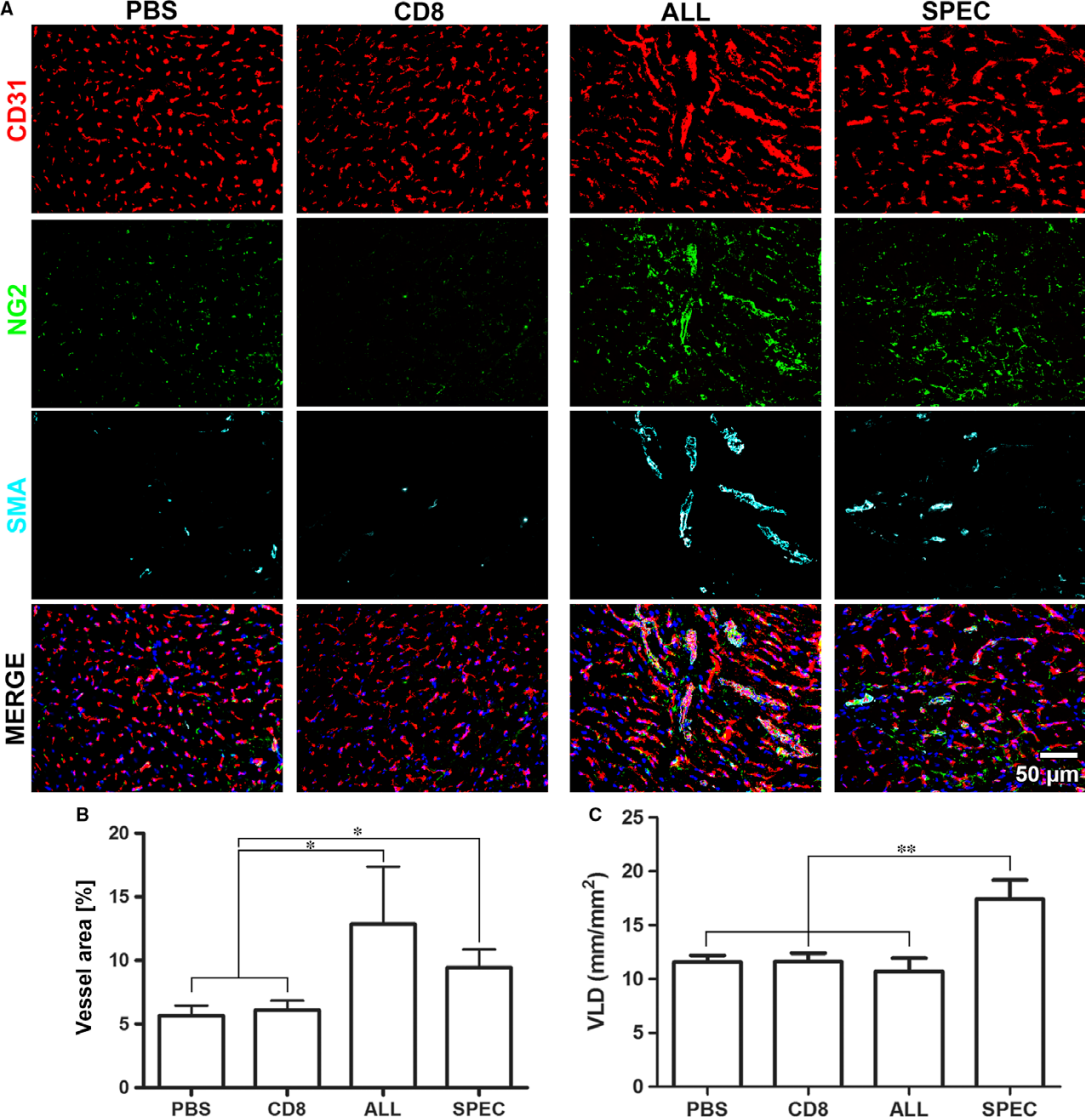
Myocardial angiogenesis. (**A**) Immunofluorescence staining for endothelium (CD31, in red), pericytes (NG2, in green), smooth muscle cells (SMA, in cyan) and nuclei (DAPI, in blue) in sections of myocardium injected with phosphate‐buffered saline, control cells (CD8) or ASC expressing uncontrolled heterogeneous levels (ALL) or a specific homogeneous VEGF level (SPEC). Angiogenesis was quantified either as the area fraction occupied by CD31‐positive pixels (**B**) or as vessel length density (**C**); *n* = 3 per group; * *P* < 0.05, ** *P* < 0.01.

The quality and quantity of vascular growth induced by controlled or heterogeneous VEGF expression were assessed by immunofluorescence staining 4 weeks after treatment. Both VEGF‐expressing populations induced robust endothelial expansion compared to PBS or control CD8 cells (Fig. 4 **A**). However, vessels induced by controlled VEGF expression (SPEC) displayed the morphology of normal and mature capillaries, homogeneous in size and associated with typical pericytes positive for the chondroitin sulphate proteoglycan NG2 and negative for smooth muscle actin (SMA) expression. These were similar in quality to the normal pre‐existing vasculature visible in the PBS and CD8 groups. A few SMA‐positive vessels of regular calibre had the morphology of arterioles feeding into the newly formed microvascular networks. In contrast, heterogeneous VEGF expression by ALL cells caused the growth of enlarged vessels, characterized by heterogeneous diameters, the presence of multiple lumens and a lack of normal pericytes, substituted by a thick coat of smooth muscle cells expressing both NG2 and SMA, similar to the progressively growing angioma‐like vascular structures previously observed in similar conditions 3, 5, 17.

Quantification of the area fraction occupied by CD31‐positive endothelial cells (Fig. 4 **B**) showed that both VEGF‐expressing populations caused a significant expansion of vascular structures compared to controls (ALL 13 ± 5% and SPEC 9 ± 1% *versus* PBS 6 ± 1% and CD8 6 ± 1%). As the vessels induced by the ALL population displayed an irregular morphology and heterogeneous sizes, vascular growth was independently quantified by measuring VLD, which indicates the total length of vessels in a given tissue area, irrespective of their diameter or endothelial cell content 5. As shown in Fig. 4 **C**, the endothelial expansion induced by uncontrolled VEGF expression did not translate into a corresponding increase in actual vessel length compared to controls (ALL 11 ± 6 mm/mm^2^ *versus* PBS 12 ± 3 mm/mm^2^ and CD8 12 ± 4 mm/mm^2^), whereas only the homogeneous VEGF‐expressing population caused a significant increase in VLD by about 60% (SPEC 17 ± 9 mm/mm^2^, *P* < 0.01 *versus* all other groups). Taken together, these data show that, although heterogeneous VEGF expression was efficient in inducing endothelial expansion, this contributed mostly to vascular enlargement rather than the generation of new capillary networks, which were instead efficiently induced by controlled expression of a homogeneous‐specific VEGF level.

### Cell engraftment and fate

**Figure 5:**
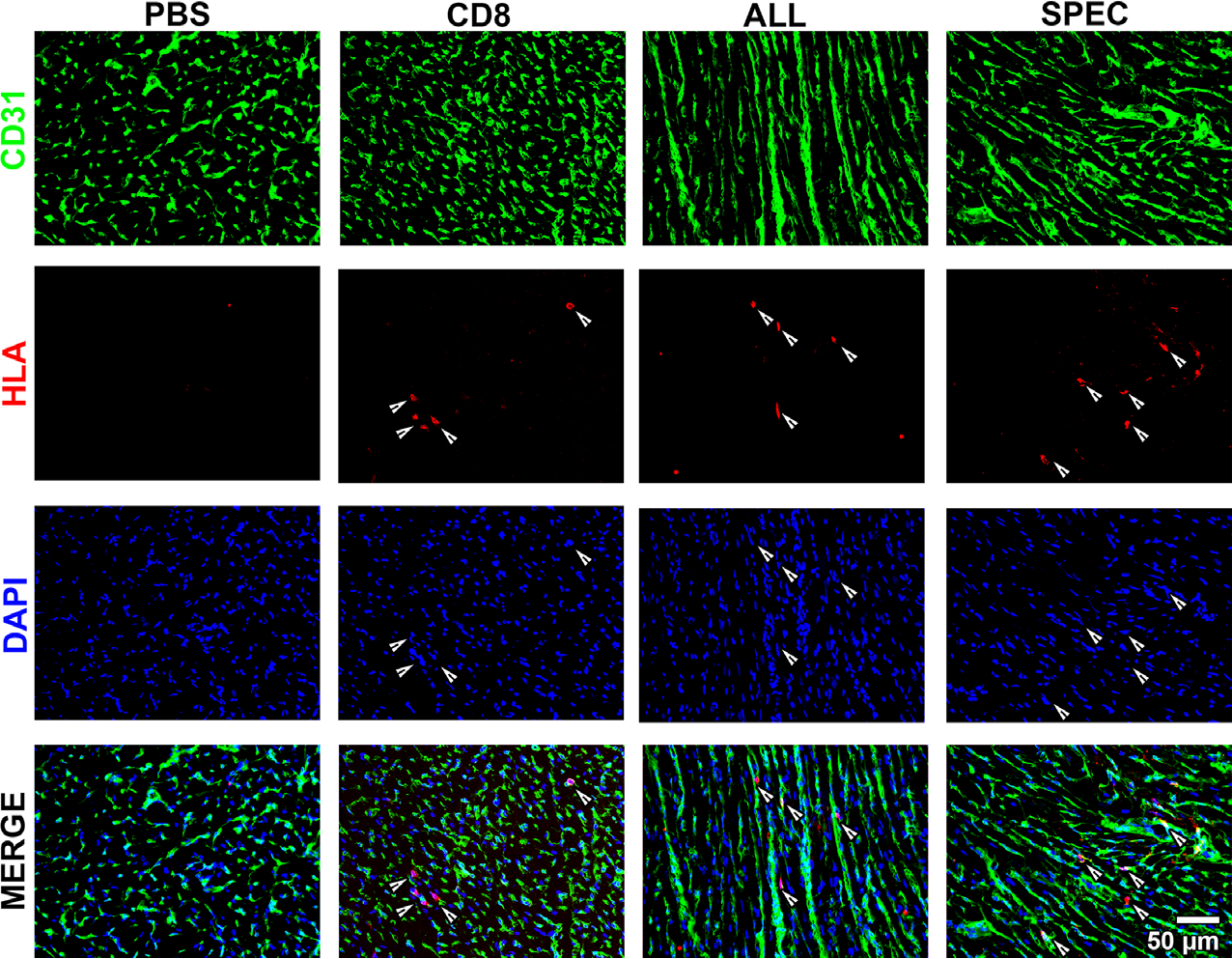
Cell engraftment. Survival of human ASC was assessed by immunostaining for (HLA)‐Class I (red), while endothelium was detected by CD31 expression (green), and nuclei by DAPI staining (blue) 4 weeks after injection of phosphate‐buffered saline, control cells (CD8) or the two VEGF ‐expressing populations (ALL and SPEC). White arrows indicate single engrafted human cells.

To determine the degree of stable engraftment of the injected cells, human ASC were tracked in harvested tissue by immunofluorescence staining for human‐specific histocompatibility antigens of class I (HLA‐class I), which are ubiquitously and specifically expressed on human cells. Four weeks after injection, only rare human ASC were still detectable in the rat myocardium (Fig. 5), which did not appear integrated with the cardiomyocytes, but rather remained as isolated single cells in between CD31‐positive vessels. No differences were observed between the three cell‐based treatment groups (CD8, ALL and SPEC), suggesting that VEGF expression did not influence the rate of ASC survival in the myocardium. Although unlikely, it remains theoretically possible that some injected cells may have down‐regulated HLA‐class I expression *in vivo*. Therefore, ASC engraftment was also analysed by *in situ* hybridization to the human‐specific Alu‐sequence, present in the genomic DNA of all human cells. This unrelated technique confirmed that ASC engraftment 4 weeks after injection was very limited, without noticeable differences between the groups (data not shown).

**Figure 6:**
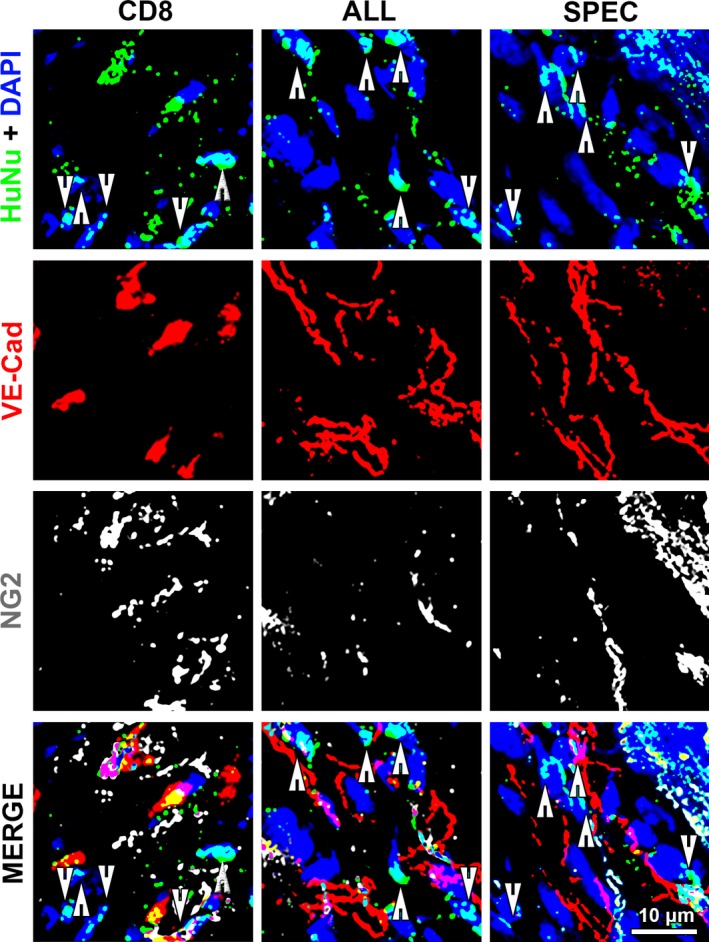
Cell fate. Fate of human ASC was assessed by immunostaining for the endothelial junctional marker VE‐Cadherin (VE‐Cad, in red), pericytes (NG2, in white), human nuclei (HuNu, in green) and nuclei by DAPI staining (blue) 4 weeks after injection of control cells (CD8) or ASC expressing uncontrolled heterogeneous levels (ALL) or a specific homogeneous VEGF level (SPEC).

Lastly, we sought to determine whether engrafted ASC might contribute to vessel growth by acquiring a vascular cell fate. For this purpose, injected ASC were identified by staining with a human nuclei antibody (HuNu), which is specific for human cells, and co‐localized with endothelial or pericyte markers (VE‐Cadherin and NG2, respectively). Most human nuclei appeared located in between vascular structures and did not co‐localize with either endothelial cells or pericytes (arrows in Fig. 6) both for the control group (CD8) and the VEGF‐expressing populations (ALL and SPEC). However, the few human cells from both VEGF‐expressing populations that were present in the myocardium 4 weeks after injection could be shown to still produce VEGF, whereas control CD8 cells did not, as expected (Fig. S2). Immunofluorescence staining showed that (*i*) the most part of rat VEGF protein was visible in association with surviving human cells, suggesting that endogenous VEGF is not significantly expressed in comparison with the exogenous delivered factor; and (*ii*) VEGF protein was tightly associated with the surface of producing cells, with no significant staining visible even a few μm away, providing a visual evidence of the concept of microenvironmental localization. Because of the essentially non‐quantitative nature of immunostaining, levels of expression by different cells could not be visualized by this technique.

## Discussion

In the present study, we have provided proof‐of‐principle that cell‐based homogeneous expression of a specific moderate VEGF level in post‐infarction myocardium is effective to (*i*) induce robust growth of normal and mature microvascular networks; (*ii*) prevent deterioration of cardiac function; and (*iii*) limit fibrotic scarring.

Myocardial ischaemia leads to thinning of the ventricle wall, myocyte slippage and ventricular dilatation, with progressive deterioration of cardiac function until end‐stage heart failure 22. Therapeutic angiogenesis, aiming at growing new blood vessels from pre‐existing vasculature through the delivery of exogenous factors, is an attractive strategy to restore blood flow in the ischaemic myocardium for many patients that cannot be treated by coronary revascularization, or as an adjunct to surgery 23. VEGF is the master regulator of both developmental and postnatal angiogenesis 24. However, despite promising results obtained in various animal models of acute or progressive ischaemia, phase I/II clinical studies based on VEGF gene therapy failed to show evidence of benefit and conflicting outcomes were reported for cardiac functionality after myocardial infarction 25. Retrospective analyses identified several issues underlying these negative results, including the low local transfection efficacy at safe vector doses, resulting in limited amount and duration of protein expression 7, 26. VEGF gene delivery by different vectors and in a variety of tissues has been shown to produce significant adverse effects, such as vascular hyperpermeability and oedema 27, exacerbated scar tissue formation 28, and aberrant endothelial cell proliferation with growth of angioma‐like tumours 4, 29. Growing evidence supports the notion that the difficulty of controlling the VEGF dose distribution in the tissue *in vivo* underlies the paradox between the crucial biological functions of VEGF and its apparent lack of a therapeutic window 8. In fact, VEGF binds to the extracellular matrix 30 and remains localized in the immediate microenvironment around each producing cell *in vivo* 5. While this property is crucial for the physiological function of VEGF to guide vascular growth through the formation of gradients in the matrix 31, it also prevents heterogeneous levels of expression from diffusing in tissue and averaging with each other 5. Therefore, the challenge is to avoid that increasing delivered dose and efficacy leads to excessive localized expression and loss of safety.

The use of stably transduced and FACS‐purified ASC can overcome this limitation, ensuring that every delivered cell produces the same dose and therefore allowing efficient and sustained VEGF expression without compromising safety. Controlled VEGF expression by the SPEC cells could induce angiogenesis that was both robust, leading to a 60% increase in vessel density, and safe, by completely preventing the appearance of aberrant vasculature. These results are in agreement with our previous results in healthy heart 17 and notably extend those findings to show that cell‐based controlled VEGF expression is safe even in ischaemic myocardium, where endogenous factors are up‐regulated in response to hypoxia and during the ensuing inflammatory reaction.

It is interesting to note that ASC expressing heterogeneous levels of VEGF completely failed to rescue cardiac functionality and, if at all, showed a non‐significant trend towards a worse outcome in contractility compared to no treatment. This observation is in agreement with previous findings in skeletal muscle that uncontrolled VEGF expression failed to improve blood perfusion and induce collateral arteriogenesis in hindlimb ischaemia 6 and likely reflects the fact that the aberrant vascular structures induced by excessive and uncontrolled VEGF doses behave as functional arterio‐venous shunts that paradoxically reduce perfusion of the downstream microcirculation despite the robust angiogenic effect 32.

The better outcomes in terms of vascularization obtained with the SPEC population likely explain the observed reduction in fibrosis and infarction stabilization. In fact, after both clinical and experimental MI, unless reperfusion is performed within 6 hours, acute and permanent coronary obstruction induces myocardial necrosis in the central infarct zone. This acute phase occurs during the first week and is associated with extensive inflammation and the recruitment of fibroblasts. On the other hand, in the border zone of the infarction, cardiomyocytes survive the acute injury, but are still critically under‐perfused and will eventually also die during the subsequent sub‐acute phase. This takes place up to 6 weeks after MI, is characterized by the gradual formation of a scar, associated with a further worsening in cardiac function and can progressively result in heart failure 20, 33. Clinical and experimental evidence 34, 35 suggest that the rarefaction of cardiac capillaries increases apoptosis of hypertrophied myocytes in the peri‐infarct region and promotes tissue hypoxia and its replacement through fibrosis. The fate of the border zone myocytes plays a key role in determining the sequelae of the acute MI, as their loss will lead to scar enlargement and worsening cardiac function, while their rescue will limit both processes. Therefore, the improved angiogenesis induced by controlled VEGF expression, delivered to the border zone during the early sub‐acute phase of MI remodelling (2 weeks after LAD ligation), is expected to be beneficial by preventing cardiomyocyte death, fibrosis expansion and decline in contractility and cardiac function over the subsequent weeks, as shown here. A central role for cardiac angiogenesis is further supported by findings that inhibition of new blood vessel formation accelerates the development of left ventricular (LV) dysfunction 36, whereas stimulation of angiogenesis improves cardiac function and delays the onset of heart failure 37, 38. Further, VEGF has been demonstrated to promote several beneficial outcomes in the cardiovascular unit, such as recruitment and activation of cardiac progenitors 39, cardiomyocyte protection from apoptosis 40 and recruitment of bone marrow‐derived mononuclear cells, which contribute to the maturation of arterial walls 41.

The observed very limited engraftment of human ASC 4 weeks after intramyocardial injection is in agreement with the previous reports 42 and has been attributed to two distinct events. First, the cell transfer is inefficient because of cell losses as a result of leakage through trans‐epicardial puncture holes, wash‐out through the venous system or squeezing of cells by the heartbeats. Second, many of the originally injected cells die because of ischaemia of the target areas and apoptosis subsequent to the loss of cell anchorage to matrix 42.

Interestingly, newly induced vessels persisted in the injection areas despite the low engraftment of implanted cells, while results in different tissues have shown that approximately 4 weeks of sustained VEGF expression are required before new vessels stabilize and can persist independently of further stimulation 5, 43. It is unlikely that this may reflect tissue‐specific differences in the stabilization kinetics of myocardial vessels, as experiments with conditional switching of VEGF expression showed that newly induced cardiac microvessels are still unstable after 2 weeks, but persist after 4 weeks 43. On the other hand, ASC have been described to secrete cytokines involved in vascular stabilization, such as transforming growth factor‐β1 (TGF‐β1) and basic fibroblast growth factor 44. Interestingly, we have recently identified a pericyte‐independent mechanism for vascular stabilization, whereby a specific subset of circulating monocytes expressing the VEGF co‐receptor Neuropilin‐1 (NEM) are recruited to sites of VEGF‐induced angiogenesis by the axon‐guidance factor Semaphorin3A and accelerate stabilization of newly formed vessels by activating endothelial TGF‐β1 signalling 45. Whether ASC may produce Semaphorin 3A and contribute to NEM recruitment remains to be investigated.

ASC can also produce angiogenic and antiapoptotic paracrine factors that contribute to cardioprotection 46. However, the findings reported here show that only VEGF overexpression at controlled levels by the SPEC population induced efficient and normal angiogenesis with functional benefit on cardiac function, whereas ASC alone did not cause any improvement compared to PBS injection, nor did any ASC population directly take part in new vessel formation as endothelial or mural cells. These controls support the conclusion that the observed functional improvements could be ascribed prevalently to the direct angiogenic effects of VEGF delivery.

The findings reported here provide proof‐of‐principle for the therapeutic potential of controlling the distribution of VEGF expression levels in the myocardium. The outcome of preventing further decline in cardiac functionality and therefore avoiding progression towards heart failure is consistent with the histological correlate of limiting the extension of fibrotic scar and is in agreement with other pre‐clinical results obtained in the same small rodent model of acute infarction, recently reviewed in a meta‐analysis by Kanelidis *et al*. 47.

In view of a potential clinical translation, the use of retroviral vectors raises some safety concerns because of the possible neoplastic transformation of transduced stem cells by insertional mutagenesis 48. However, the same approach is readily applicable to progenitors transduced with clinically compliant new‐generation vectors, such as self‐inactivating lentiviruses with chromatin insulator elements that ensure stable and sustained transgene expression in the absence of oncogenic potential 49.

In conclusion, we obtained functional evidence that controlled VEGF expression by transduced and FACS‐purified ASC is safe and effective in inducing robust growth of uniformly normal blood vessels in ischaemic myocardium, preventing the loss of cardiac functionality and reducing heart fibrosis after myocardial infarction. These results suggest that this cell‐based gene delivery approach, by affording precise control over the distribution of expression levels *in vivo*, could help overcome a significant obstacle towards safe and effective therapeutic angiogenesis in the heart.

## Conflict of interest

No competing financial interests exist.

## Supporting information


**Figure S1** Cell morphology. Before *in vivo* injection, cell morphology was assessed by fluorescence microscopy after *in vitro* staining for cell surface CD8a expression for control cells (CD8) or the two VEGF‐expressing populations (ALL and SPEC). Size bar: left column = 500 μm, right column = 50 μm.

**Figure S2** VEGF production. Production of rat VEGF by the injected human cells (control cells CD8; VEGF‐producing cells ALL and SPEC) after 4 weeks *in vivo* assessed by immunostaining for rat VEGF (rVEGF, in red), human nuclei (HuNu, in green) and nuclei by DAPI staining (blue).
