# Supplementary figures and images for: Myocardial infarction stabilization by cell‐based expression of controlled Vascular Endothelial Growth Factor levels
Source: J Cell Mol Med. 2018 Feb 25;22(5):2580–91. doi: 10.1111/jcmm.13511 (PMC5908097; doi:10.1111/jcmm.13511)

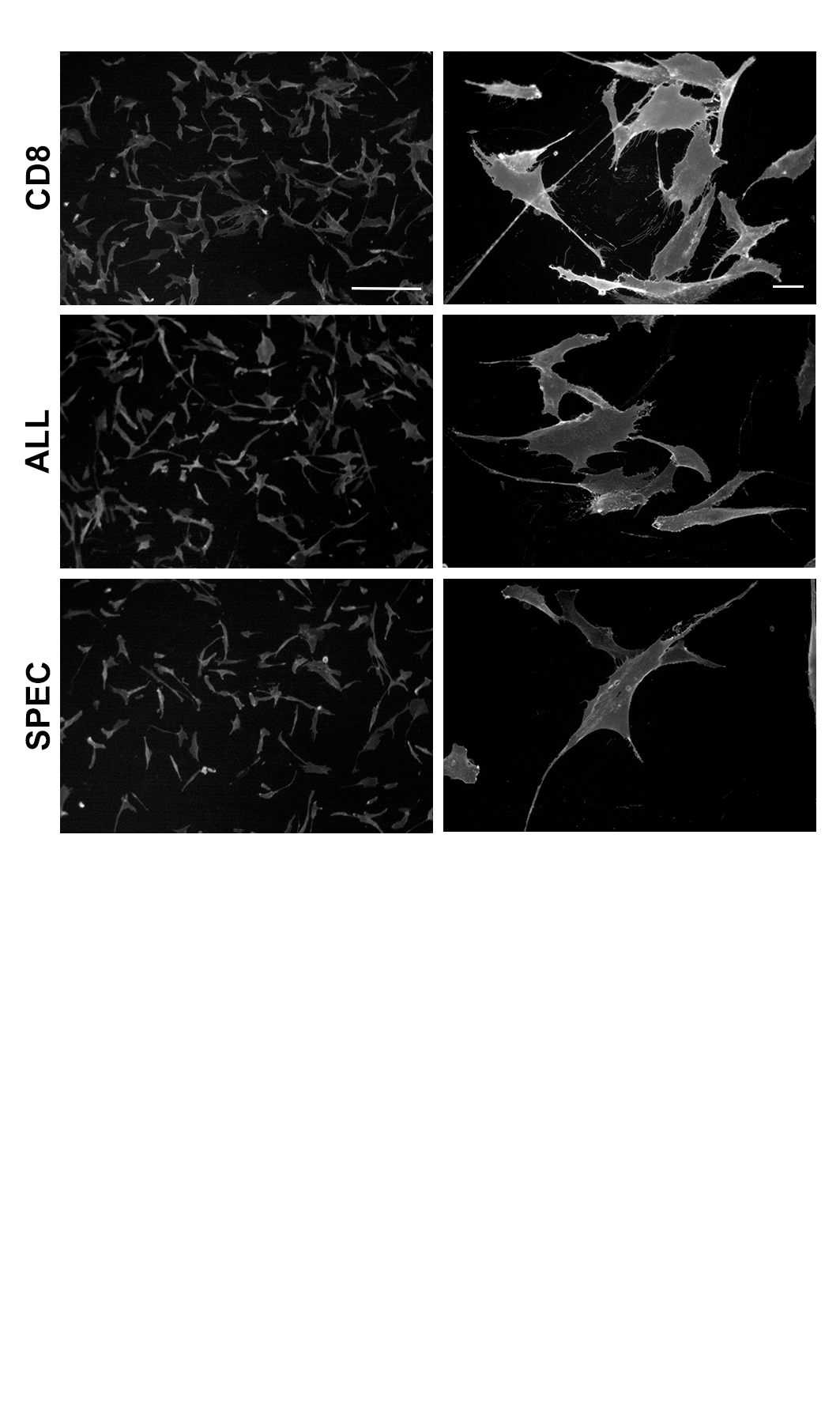

Supplement: Supplementary file 1 — Figure S1 Cell morphology. Before in vivo injection, cell morphology was assessed by fluorescence microscopy after in vitro staining for cell surface CD8a expression for control cells (CD8) or the two VEGF‐expressing populations (ALL and SPEC). Size bar: left column = 500 μm, right column = 50 μm. [file JCMM-22-2580-s001.tif]

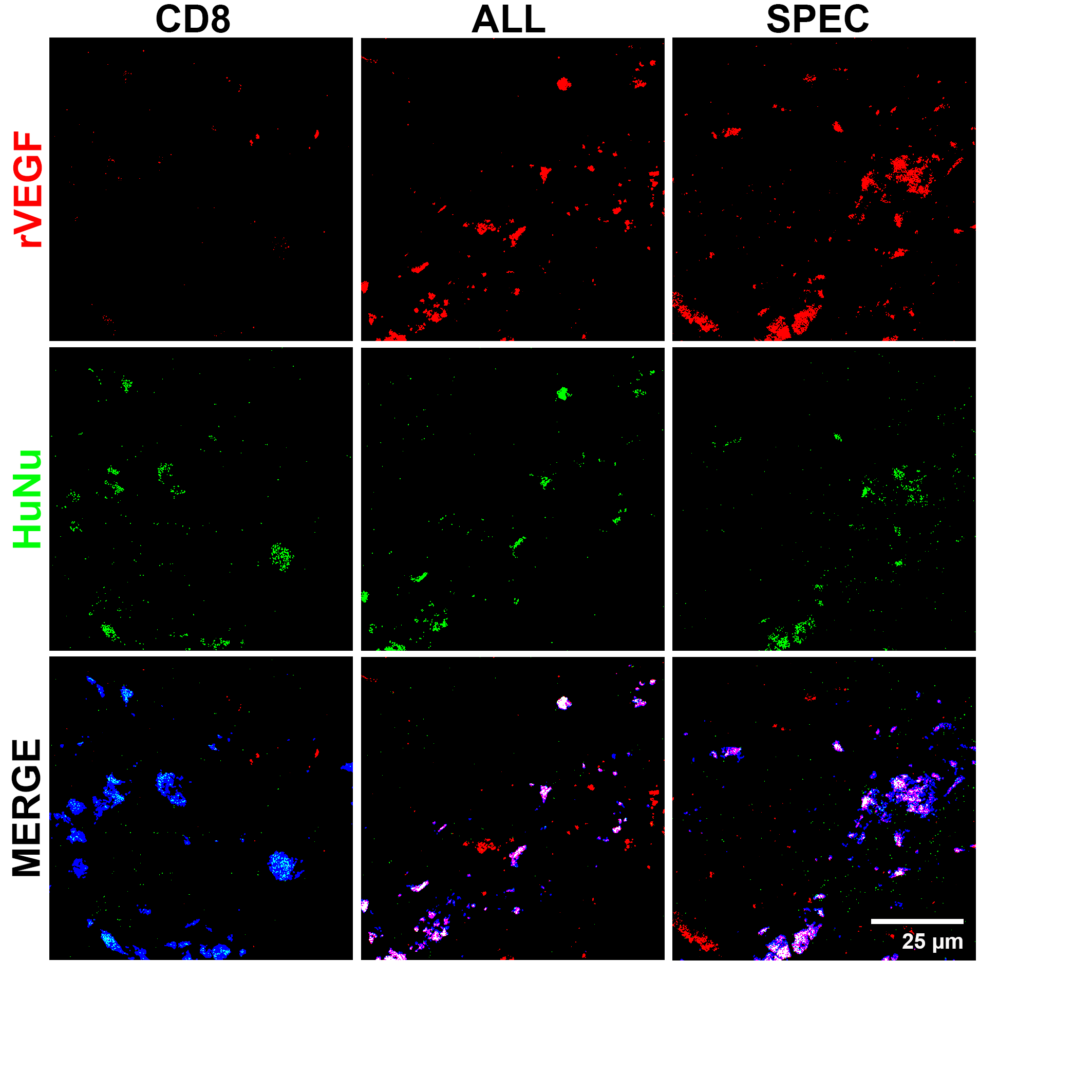

Supplement: Supplementary file 2 — Figure S2 VEGF production. Production of rat VEGF by the injected human cells (control cells CD8; VEGF‐producing cells ALL and SPEC) after 4 weeks in vivo assessed by immunostaining for rat VEGF (rVEGF, in red), human nuclei (HuNu, in green) and nuclei by DAPI staining (blue). [file JCMM-22-2580-s002.tiff]
